# Acceptance of Allogeneic Serum Eye Drops Among Users of Autologous Serum Eye Drops—A Survey-Based Study

**DOI:** 10.3390/jcm15176537

**Published:** 2026-08-24

**Authors:** Frank Blaser, Mohammadjavad Abbasi, Mario D. Toro, Timothy Hamann, Daniel Barthelmes, Sandrine A. Zweifel, Martina M. Bosch, Isabelle Meneau, Jana Schneider, Sadiq Said, Vinoth Yogarasa

**Affiliations:** 1 Department of Ophthalmology, University Hospital Zurich, University of Zurich, 8091 Zurich, Switzerland; timothy.hamann@usz.ch (T.H.); daniel.barthelmes@usz.ch (D.B.); sandrine.zweifel@usz.ch (S.A.Z.); isabelle.meneau@usz.ch (I.M.); jana.schneider@usz.ch (J.S.); sadiq.said@usz.ch (S.S.); 2 School of Medicine and Surgery, University of Naples Federico II, 80131 Naples, Italy; mj.abbas@live.com; 3 Public Health Department, University of Naples Federico II, 80131 Naples, Italy; mariodamiano.toro@unina.it; 4 Augenklinik Wettingen, 5430 Wettingen, Switzerland; martina.knecht-boesch@augenklinik-baden.ch (M.M.B.); vinoth-1996@hotmail.com (V.Y.)

**Keywords:** autologous serum eye drops, allogeneic serum eye drops, ocular surface disease, dry eye, survey

## Abstract

**Background/Objectives:** To assess the acceptance of, attitudes towards, and concerns regarding allogeneic serum eye drops (Allo-SEDs) among patients treated with autologous serum eye drops (ASEDs). **Methods:** We conducted this investigator-initiated, cross-sectional, single-center survey at the Department of Ophthalmology, University Hospital Zurich, Switzerland. We consecutively recruited all patients using ASEDs during routine visits, who anonymously completed a 9-item questionnaire comprising eight 11-point Likert-scale items and one open numerical item on the daily application frequency. We assessed perceived ASED effectiveness, willingness to switch to Allo-SEDs under different scenarios, and concerns regarding safety, efficacy, and immunological risks. **Results:** Of 202 eligible patients, 107 returned the questionnaire (53.0%). The full eligible cohort had a median (IQR) age of 63.5 (49.2–75.0) years and a median ASED treatment duration of 4.0 (1.0–8.0) years. They rated ASEDs as highly effective (median 9, IQR 8.2–10.0) and applied them a median of 7 (5–10.5) times daily. Willingness to switch was low without clinical need (median 3, IQR 0–6; *p* = 1.0), increased when autologous production was no longer feasible (median 7, IQR 5–9; *p* < 0.001), and was intermediate for general openness (median 5, IQR 2–8; *p* = 0.7). Concerns regarding infectious disease transmission, immunological reactions, and therapeutic efficacy correlated with lower willingness to switch (all r = −0.29 to −0.46). Daily application frequency did not correlate with acceptance. **Conclusions:** Patients were highly satisfied with ASEDs but were reluctant to switch to Allo-SEDs unless autologous production was no longer feasible. Among the domains assessed, concerns regarding infection, immunological reactions, and efficacy were the main barriers to acceptance, supporting targeted patient education.

## 1. Introduction

Severe Ocular Surface Diseases (OSDs) affect millions of people worldwide, cause significant ocular morbidity, and significantly reduce quality of life [1,2]. Dry eye disease (DED) is one of the most common forms of OSD, with reported prevalence estimates of approximately 10–30% [2] and a substantial patient-reported burden across Europe [3]. As a result, severe OSDs are recognized as a significant public health and socioeconomic challenge, underscoring the need for effective treatment options [4].

Serum eye drops (SEDs) are blood-derived tear substitutes used as a second- or third-line treatment for OSD after conventional therapy with topical lubrication, anti-inflammatory treatment, or punctum plugs has failed [5]. Two different types of SEDs are differentiated. While autologous SEDs (ASEDs) are prepared from a patient’s own blood, allogeneic SEDs (Allo-SEDs) are derived from a donor’s blood after initial screening for infectious diseases [6]. Donor selection for Allo-SEDs prioritizes ABO compatibility to minimize immunological reactions [7]. Group AB donors are often preferred because their serum lacks anti-A and anti-B antibodies, making it universally compatible across all blood groups. Further, male donors without a transfusion history are favored to reduce the risk of human leukocyte antigen (HLA) antibodies, which are more common in females due to pregnancy-related alloimmunization and in transfused individuals [7,8]. Production protocols vary internationally and may utilize single-donor serum or pooled serum from a limited number of compatible donors, followed by aliquoting into individual vials [9,10,11]. The manufacturing processes for ASEDs and Allo-SEDs follow the same basic principles. Blood is collected without anticoagulant, allowed to clot, and centrifuged to separate the serum from the cellular components without hemolysis. The serum is decanted aseptically, frequently filter-sterilized (0.2 µm), and, where required, diluted with a preservative-free vehicle such as balanced salt solution or saline, most often to a concentration of 20–50%, although higher concentrations up to undiluted serum are also used [9,11,12]. The drops are then filled into applicators and stored at −20 °C, which allows a shelf life of up to three to six months, whereas the vial in use is kept refrigerated [12,13]. The two preparations therefore differ less in the manufacturing process itself than in the origin of the blood and in the donor screening that precedes it. To date, no internationally harmonized protocol for the preparation of SEDs exists, although several groups have proposed consensus protocols to improve quality, safety, and consistency [10,14]. Unlike regular eye drops, SEDs not only lubricate the eye but also contain multiple biologically active growth factors, vitamins, and further nutrients that promote epithelial healing and support ocular surface homeostasis [12]. Typical indications for the use of SEDs include neurotrophic keratopathy, severe OSD or DED, persistent epithelial defects, ocular graft-versus-host disease, and recurrent corneal erosions [15].

Given these shared indications, a key clinical question is whether autologous and allogeneic SEDs differ in effectiveness and safety. Several studies have shown that there is no significant clinical disparity regarding the effectiveness of autologous versus allogeneic SEDs [11,16]. Both types similarly improved patients’ OSD index scores, reduced patient-reported symptoms, and were equally tolerated. Furthermore, both showed improved tear film stability (i.e., increased tear breakup time), reduced corneal staining, and increased goblet cell density [17,18]. They both share similar biochemical properties and have the same therapeutic principles [13].

However, they differ fundamentally in their sources, safety profiles, immunological responses, and availability. ASEDs have the advantage of complete immunological compatibility, eliminating the risk of immune-mediated reactions [19]. However, they may be unsafe in patients with anemia, cardiovascular disease, or limited venous access [16]. Allo-SEDs reduce these limitations but carry the risk of infectious disease transmission and an increased risk of immunological reactions [14]. In selected systemic autoimmune or inflammatory conditions, however, Allo-SEDs may even be theoretically more advantageous, as autologous serum can contain circulating autoantibodies and pro-inflammatory mediators that are largely absent in donor serum. In line with this concept, improved clinical outcomes have been reported in patients with ocular graft-versus-host disease treated with allogeneic serum eye drops [20]. Furthermore, Allo-SEDs usually have lower waiting times because ready-made drops are available off-the-shelf [16]. Allo-SEDs also offer greater standardization, as donor blood serum can be processed to achieve consistent concentrations of key components [7]. ASED compositions, on the other hand, tend to vary, as serum component concentrations differ from patient to patient [14]. This standardization advantage of Allo-SEDs is a key factor in why some healthcare systems, such as in Australia or New Zealand, have adopted allogeneic drops as a preferred option [6,21]. In the Netherlands too, Allo-SEDs have effectively replaced autologous production and are supplied as a centrally manufactured, pooled, off-the-shelf product, with the treating ophthalmologists guiding patients through the transition from ASEDs [16,22]. Worldwide, autologous serum eye drops remain the predominant standard of care. ASEDs have been historically preferred due to their aforementioned immunological safety and personalized nature [23].

This survey-based study aims to investigate the acceptance of allogeneic SEDs among patients currently treated with autologous SEDs at the University Hospital Zurich. By administering a questionnaire, we sought to assess patients’ attitudes, preferences, and concerns regarding the potential use of donor-derived SEDs. Understanding patient perspectives is essential when considering changes to established treatment protocols, particularly when clinical efficacy is comparable, but safety profiles and practical considerations differ. At present, eight manufacturers in Switzerland hold a Swissmedic authorization for their respective ASED manufacturing process, whereas no manufacturer holds such an authorization for Allo-SEDs. The results of this study may support clinicians and stakeholders in understanding patient perspectives and may serve as a reference for future considerations regarding serum-based therapies in Switzerland.

## 2. Materials and Methods

This investigator-initiated, cross-sectional, survey-based, single-center study was conducted at the Department of Ophthalmology of the University Hospital Zurich, Switzerland. The study aimed to assess patients’ acceptance, attitudes, and concerns regarding the potential use of allogeneic SEDs among individuals currently treated with autologous SEDs. We distributed the survey to patients applying ASEDs during routine outpatient visits. The responsible local ethics committee reviewed the study protocol and declared no objection (BASEC Req-Nr. 2026-00134). The study adhered to the tenets of the Declaration of Helsinki. Participation was voluntary, and we obtained verbal informed consent from all participants before study inclusion. We handled all data in accordance with Good Clinical Practice guidelines.

We consecutively recruited all patients regularly using ASEDs for various clinical indications during routine outpatient visits. Patients received a neutral verbal explanation and standardized instructions before being handed the questionnaire together with a pre-stamped return envelope. No exclusion criteria were applied based on underlying diagnosis, treatment duration, or application frequency. We retrieved all demographic and clinical characteristics—age, sex, underlying diagnosis, duration of ASED treatment, and prescribed serum concentration—from the electronic medical records of the full eligible cohort of 202 patients. Participants returned the questionnaires anonymously and without any identifier, so that we could not link the responses to individual clinical records. These characteristics therefore describe the full eligible cohort and not the 107 respondents, and a comparison between responders and non-responders was not possible. The survey consisted of a 9-item questionnaire developed specifically for this study and administered in the participants’ native language (German) to ensure optimal comprehension and clarity. The questionnaire items were based on the existing literature on SEDs and allogeneic blood products and refined on the basis of our clinical experience to capture the factors considered most relevant to patient acceptance. The full original questionnaire is provided in Supplementary File S1, and the English translation of the questionnaire is presented in Table 1. Eight items used 11-point Likert scales anchored at 0 (does not apply at all), 5 (neutral), and 10 (fully applies), whereas the item on the daily application frequency asked for an open numerical value, which we analyzed as a quantitative count variable. The questionnaire assessed, in the order presented to participants, the perceived effectiveness of ASEDs, their daily frequency of use, the importance of trust in the safety and quality control of SED preparations in general, the willingness to switch from ASEDs to Allo-SEDs across three predefined scenarios: (i) a voluntary switch in the absence of any clinical need (Q4 Voluntary), (ii) a switch necessitated because autologous production was no longer possible (Q5 Forced), for example owing to a regulatory or manufacturing decision to discontinue autologous preparation or to patient-specific contraindications to repeated blood donation, and (iii) a general openness toward Allo-SED preparations (Q6 Consider), and concerns regarding infectious disease transmission, immunological reactions, and reduced therapeutic efficacy. The data were retrieved anonymously.

Descriptive statistics were computed for all variables. Continuous data are presented as means with standard deviations (SDs) and medians with interquartile ranges (IQRs) or ranges, as appropriate. Categorical variables are summarized as frequencies and percentages. The statistical analyses were performed using R version 4.2.2 (R Foundation for Statistical Computing, Vienna, Austria). All figures were generated using the ggplot2 and corrplot packages within the R (version 4.2.2) environment. Due to the ordinal nature of the Likert-scale responses and significant deviations from normality confirmed by Shapiro–Wilk tests (*p* < 0.001 for all items), non-parametric tests (Wilcoxon signed-rank, Mann–Whitney U, and Spearman correlation) were used throughout. A two-sided *p*-value of <0.05 was considered statistically significant, except for paired comparisons where a Bonferroni correction was applied for multiple testing.

## 3. Results

### 3.1. Study Population and Treatment Characteristics

From November 2025 to March 2026, we identified 202 patients regularly applying ASEDs, of whom 107 (53.0%) returned the questionnaire and were included in the final survey analysis. Of the 202 patients, 136 (67.3%) were female. The median (IQR, range) age was 63.5 (49.2 to 75.0, 9 to 94) years, and the median (IQR, range) duration of ASED treatment was 4.0 (1.0 to 8.0, 0.0 to 21.0) years. Regarding the serum concentration, 140 of 202 (69.3%) patients applied 1:1 diluted (with balanced salt solution) ASEDs, whereas 62 of 202 (30.7%) received undiluted preparations. Table 2 provides an overview of the patient characteristics. The underlying indications were heterogeneous, with the most common diagnoses being dry eye disease (42.1%), Sjögren’s syndrome (13.9%), and graft-versus-host disease (9.9%). Neurotrophic keratopathy of various etiologies, including herpes simplex virus (4.5%), herpes zoster ophthalmicus (4.0%), and unspecified causes (4.0%), represented further indications, alongside corneal neuropathic pain (2.5%), post-refractive surgery (2.5%), limbal stem cell deficiency (2.0%), and chemical burns (2.0%). Further, rare conditions included Stevens–Johnson syndrome (SJS), mucous membrane pemphigoid (MMP), and toxic epidermal necrolysis (TEN). When stratified by serum concentration, conditions such as Sjögren’s syndrome (85.7%) and graft-versus-host disease (85.0%) were predominantly treated with diluted serum, whereas patients with neurotrophic keratopathy after herpes simplex virus (55.6%) and toxic epidermal necrolysis (80.0%) more frequently received undiluted serum. Table 3 shows the primary diagnoses and the prescribed ASED concentrations in more detail.

### 3.2. Perceived Effectiveness, Application Frequency, and Importance of Trust in SED’s Therapy (Survey Questions 1 to 3)

**Figure 1:**
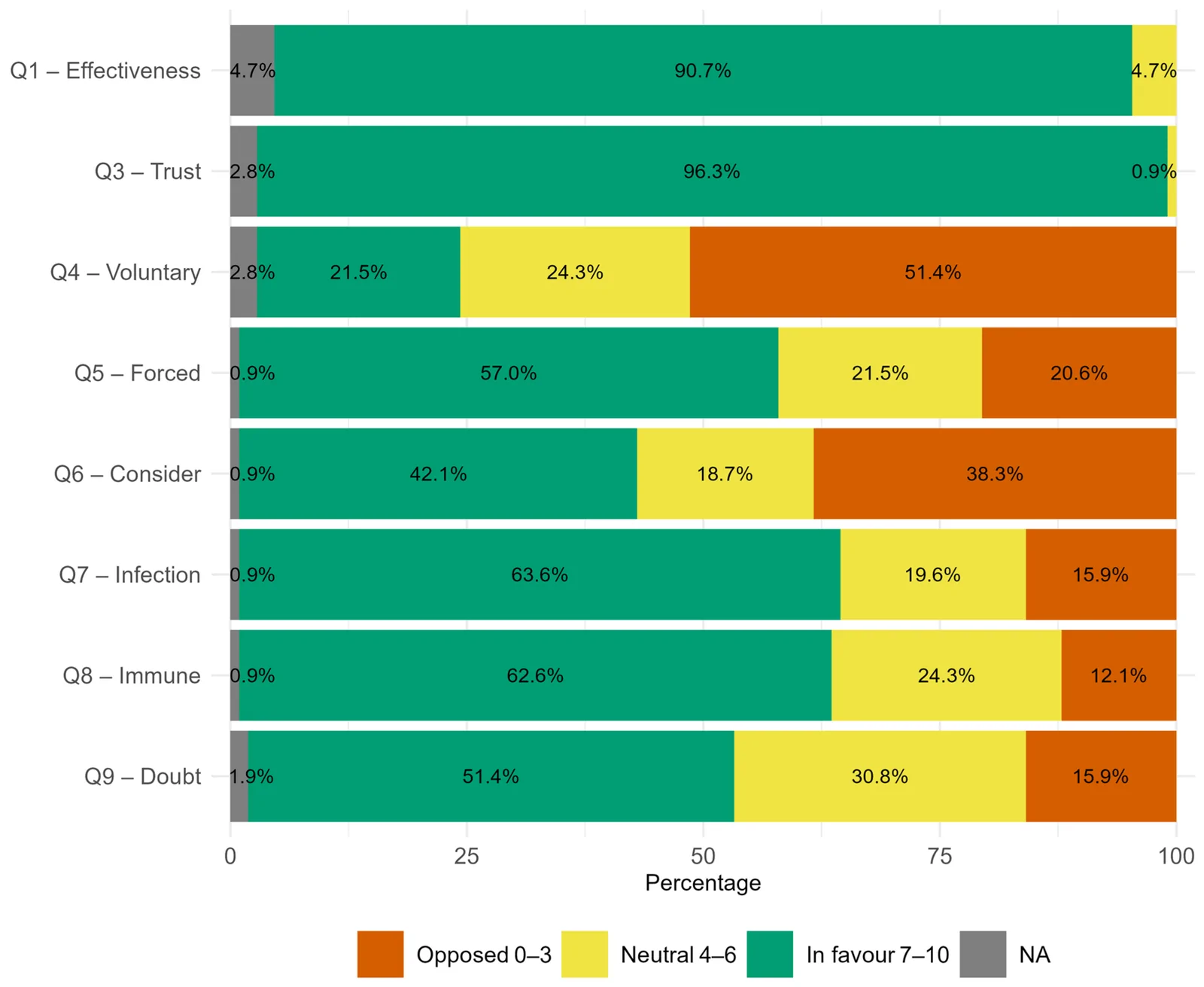
Distribution of responses to the Likert-scale items. The stacked bars chart shows the percentage of participants selecting each score from 0 (does not apply at all/very poor) to 10 (fully applies/very good) for the eight 11-point items: perceived effectiveness of current autologous serum eye drops (Effectiveness, Q1), importance of trust in the safety and quality control of serum eye drops (Trust, Q3), willingness to switch voluntarily in the absence of a clinical need (Voluntary, Q4), willingness to switch when autologous production is no longer feasible (Forced, Q5), general openness to allogeneic preparations (Consider, Q6), and concerns regarding infectious disease transmission (Infection, Q7), immunological reactions (Immune, Q8), and reduced therapeutic efficacy (Doubt, Q9). The study questions are displayed in Table 1.

**Figure 2:**
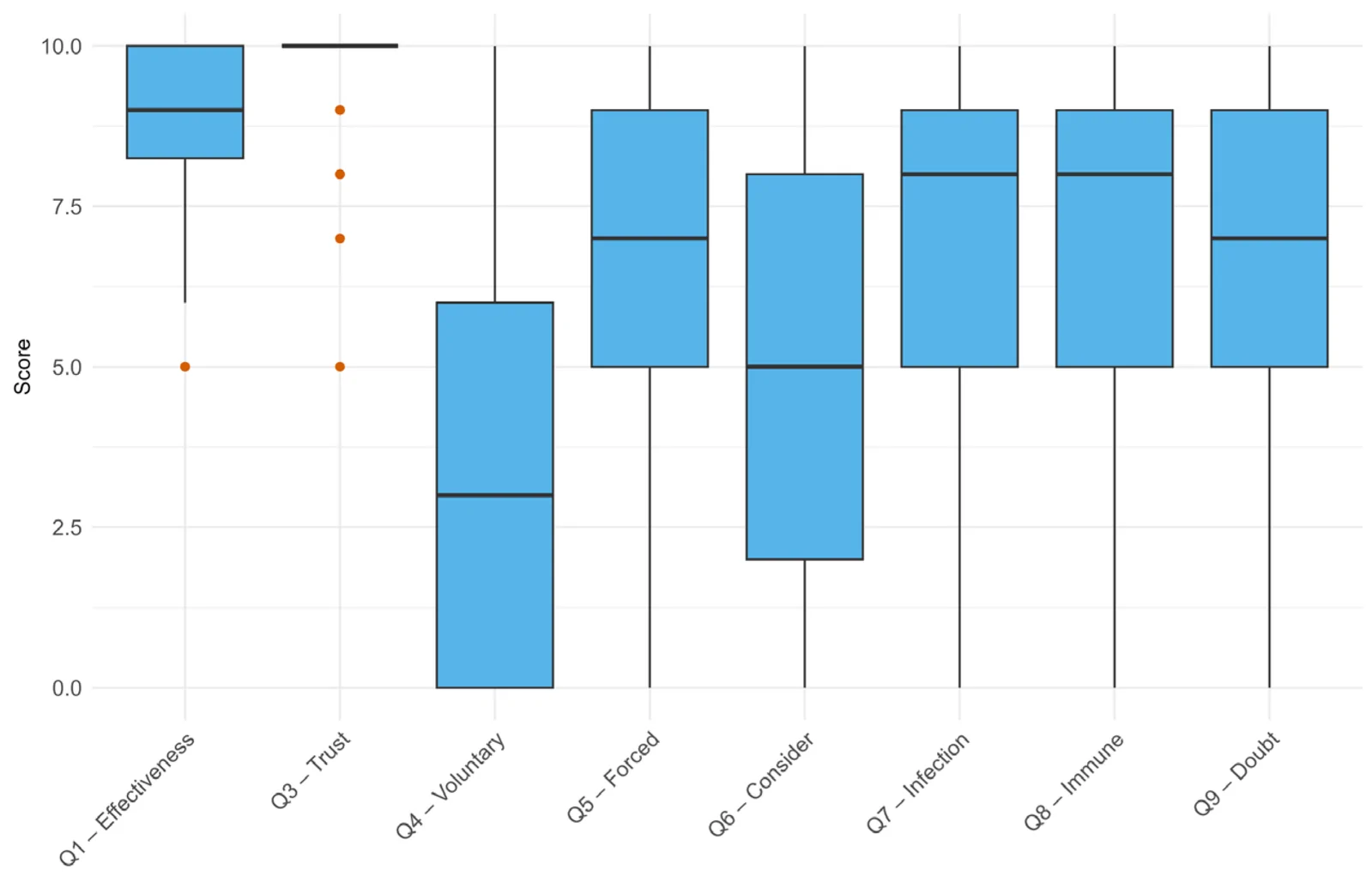
Summary of score distributions for all eight Likert-scale items, displayed as boxplots. Items are shown along the x-axis in survey order: perceived effectiveness of current autologous serum eye drops (Effectiveness, Q1), importance of trust in safety and quality control (Trust, Q3), willingness to switch voluntarily in the absence of clinical need (Voluntary, Q4), willingness to switch when autologous production is no longer feasible (Forced, Q5), general openness to allogeneic preparations (Consider, Q6), and concerns regarding infectious disease transmission (Infection, Q7), immunological reactions (Immune, Q8), and reduced therapeutic efficacy (Doubt, Q9). Scores range from 0 (does not apply at all/very poor) to 10 (fully applies/very good). Red dots represents outliers, defined as values beyond 1.5xIQR from the first or third quartile.

Participants reported a high perceived effectiveness of their current ASED treatment, with a median (IQR) score of 9 (8.2 to 10.0). The patients applied ASEDs a median (IQR) of 7 (5 to 10.5) times per day. Further, they considered trust in the safety and quality control of SEDs highly important, assigning a median (IQR) score of 10 (10 to 10). Figure 1 and Figure 2 display the responses to all Likert-scale items.

### 3.3. Willingness to Switch to Allo-SEDs (Survey Questions 4 to 6)

**Figure 3:**
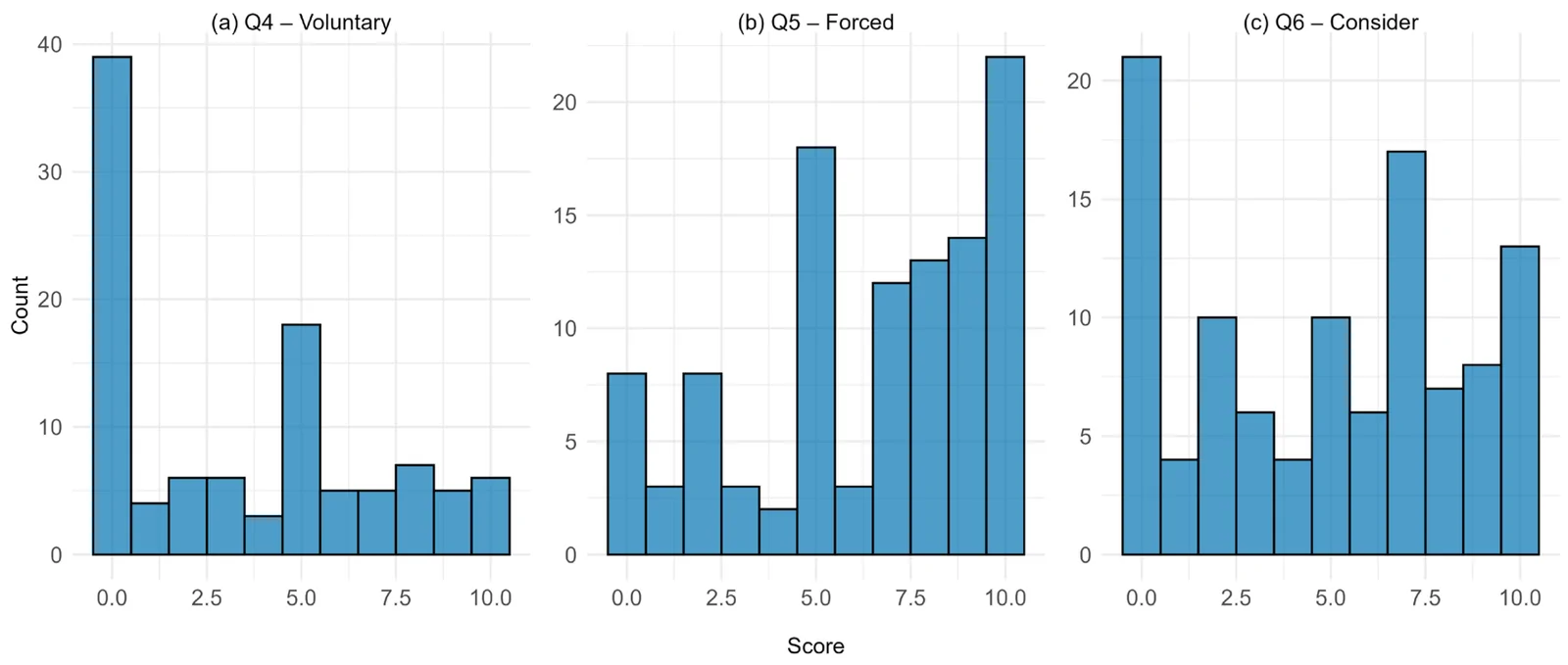
Distribution of the willingness-to-switch scores across the three predefined scenarios: (**a**) switching voluntarily in the absence of a clinical need (Voluntary, Q4); (**b**) switching when autologous production is no longer feasible (Forced, Q5); and (**c**) general openness to allogeneic preparations (Consider, Q6). Histograms show the frequency of each response from 0 to 10, with a score of 5 representing neutrality. The study questions are displayed in Table 1.

**Figure 4:**
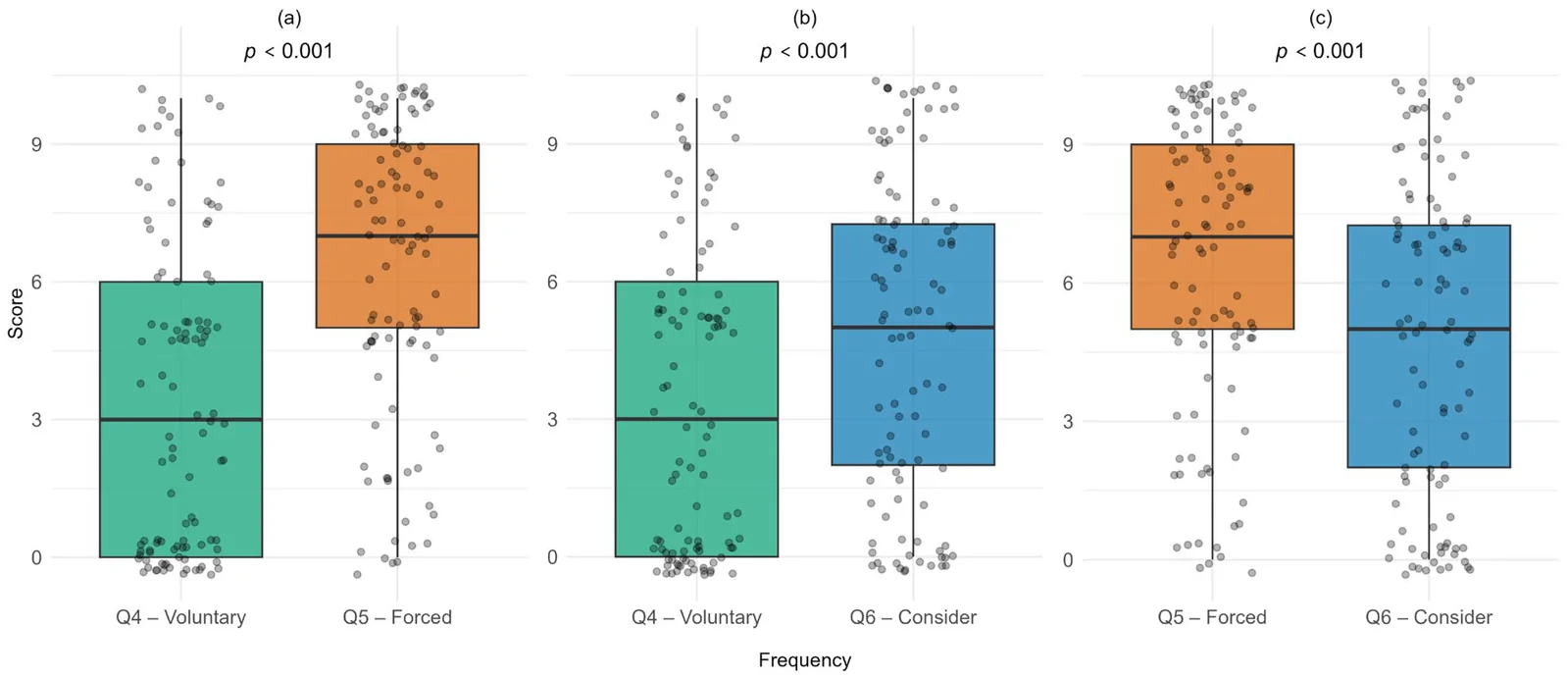
Pairwise comparisons of the willingness-to-switch scores between the three scenarios. Paired plots show each participant’s scores for (**a**) Voluntary (Q4) versus Forced (Q5); (**b**) Voluntary (Q4) versus Consider (Q6); and (**c**) Forced (Q5) versus Consider (Q6). All three pairwise comparisons were statistically significant (*p* < 0.001; Wilcoxon signed-rank test). The study questions are displayed in Table 1. In each panel, boxplots are color-coded: green = Voluntary (Q4), orange = Forced (Q5), and blue = Consider (Q6).

We assessed the willingness to switch from ASEDs to Allo-SEDs across three predefined scenarios: switching voluntarily in the absence of a clinical need, switching when autologous production is no longer feasible, and general openness to allogeneic preparations. Regarding the willingness to switch, we used one-sided Wilcoxon signed-rank tests to analyze Likert scales from 0 to 10, with a score of 5 representing neutrality. Voluntary willingness to switch (survey question 4) in the absence of a clinical need was low, with a median (IQR, 95% CI) score of 3 (0 to 6, 1.0 to 5.0), not significantly exceeding the neutral midpoint of 5 (*p* = 1.0, one-sided Wilcoxon test for superiority over 5; the median is significantly below 5 in a two-sided test, *p* < 0.001). Regarding the willingness to switch in case autologous production was no longer feasible (e.g., due to anemia or difficult venous access; survey question 5), we found a median (IQR, 95% CI) of 7 (5 to 9, 6 to 8), significantly exceeding the neutral midpoint of 5 (*p* < 0.001, effect size *r* = 0.4). Hypothetical openness to generally consider Allo-SEDs (survey question 6) yielded a median (IQR, 95% CI) of 5 (2 to 8, 4 to 7) and did not significantly differ from the neutral midpoint (*p* = 0.700). All three pairwise comparisons (i.e., voluntary versus forced, voluntary versus openness to switch, and forced versus openness to switch) reached statistical significance (*p* < 0.001, respectively). Figure 3 shows the score distributions of the three scenarios and Figure 4 the corresponding pairwise comparisons.

### 3.4. Concerns Regarding Allo-SEDs (Survey Questions 7 to 9)

Concerns regarding Allo-SEDs were pronounced across all three assessed domains, with median (IQR) scores of 8 (5 to 9) for infectious disease transmission, 8 (5 to 9) for immunological reactions, and 7 (5 to 9) for therapeutic efficacy doubts. One-sided Wilcoxon signed-rank tests confirmed that each concern score was significantly elevated above the neutral midpoint of 5 (Q7: *p* < 0.001; Q8: *p* < 0.001; Q9: *p* < 0.001). The three concern items are displayed in the overall Likert-scale distribution shown in Figure 1.

### 3.5. Associations Between Application Frequency, Concerns, and Acceptance of Allo-SEDs

To investigate whether daily application frequency influenced acceptance of Allo-SEDs, we calculated Spearman rank correlations between the reported daily number of ASED applications (Q2) and each of the three acceptance scores (Q4–Q6). None of the correlations reached statistical significance: voluntary (Q4): ρ = 0.14, 95% CI –0.06 to 0.34, *p* = 0.161; forced (Q5): ρ = 0.04, 95% CI –0.15 to 0.23, *p* = 0.681; openness to switch (Q6): ρ = 0.06, 95% CI –0.13 to 0.25, *p* = 0.542. These findings confirm that daily application frequency does not correlate with willingness to switch to Allo-SEDs. The corresponding subgroup analysis based on dichotomised frequency groups is provided in the Supplementary Material.

**Figure 5:**
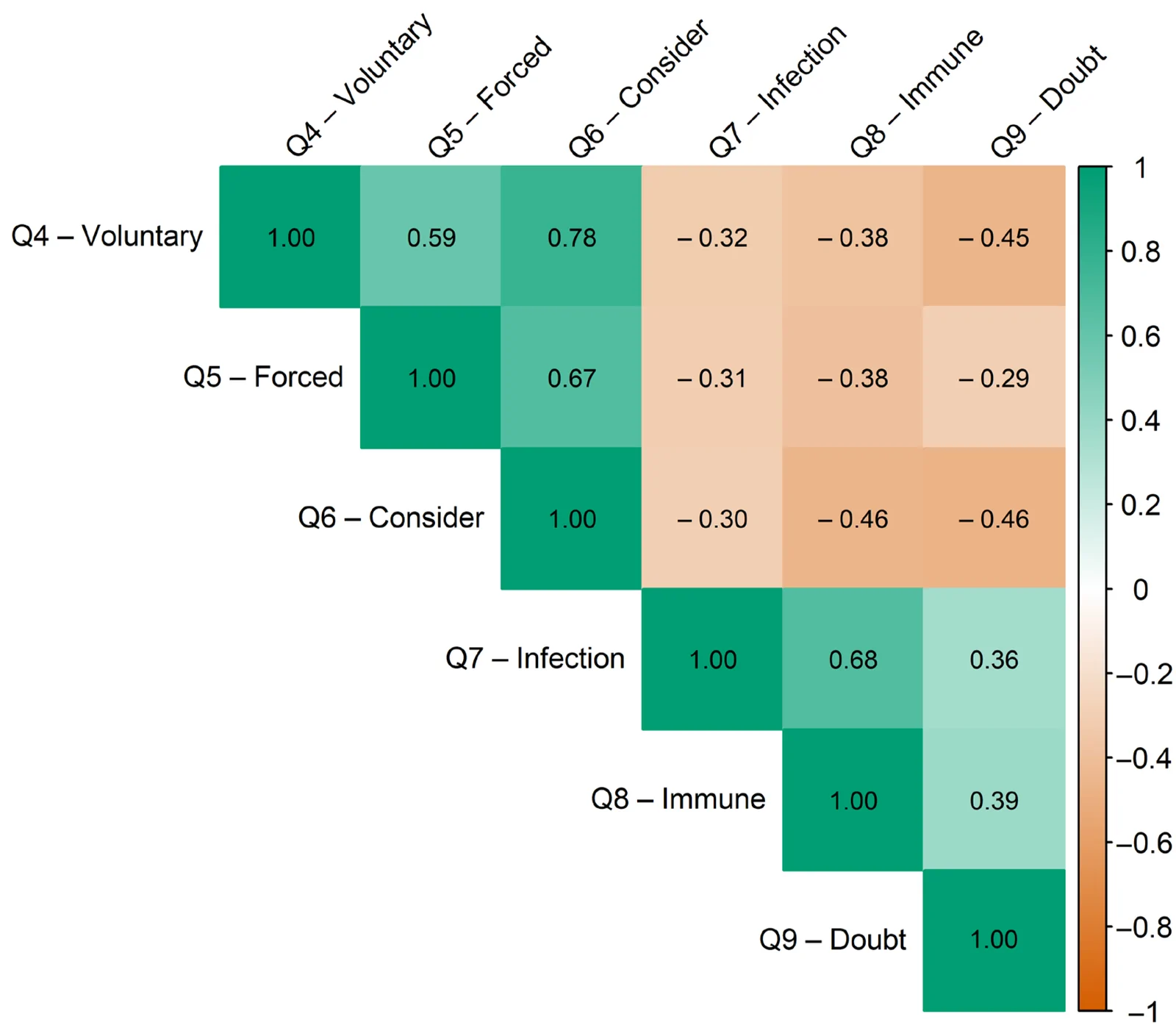
Spearman rank correlation matrix of the six 11-point items covering acceptance of and concerns regarding allogeneic serum eye drops. Each cell displays the correlation coefficient (ρ). The color scale encodes the direction and strength of the association, with brown indicating negative and blue-green indicating positive correlations. The three acceptance items correlated positively with one another (ρ = 0.59 to 0.78), as did the three concern items (ρ = 0.36 to 0.68). All nine correlations between concern and acceptance items were negative (ρ = −0.29 to −0.46), showing that participants with stronger concerns reported a lower willingness to switch. All nine correlations remained statistically significant after Bonferroni correction (α = 0.05/9 = 0.006).

To assess whether concerns about Allo-SEDs were associated with the willingness to switch, we calculated Spearman rank correlations between each concern item (survey questions 7 to 9) and each acceptance item (survey questions 4 to 6). All nine pairwise correlations were negative, indicating that greater concerns were consistently associated with lower acceptance, with correlation coefficients ranging from −0.29 to −0.46 and confidence intervals excluding zero throughout (Figure 5). To account for multiple comparisons, we applied a Bonferroni correction (α = 0.05/9 = 0.006); all nine correlations remained statistically significant. The strongest correlations emerged between concerns about immunological reactions and the general openness to Allo-SEDs (ρ = −0.46, 95% CI −0.61 to −0.29), as well as between doubts regarding therapeutic efficacy and the same item (ρ = −0.46, 95% CI −0.65 to −0.26).

## 4. Discussion

This study investigated the acceptance, attitudes, and concerns regarding allogeneic SEDs among patients currently treated with autologous SEDs at the University Hospital Zurich. The majority of participants demonstrated low willingness to switch voluntarily to Allo-SEDs in the absence of clinical necessity, while acceptance increased substantially when autologous production was hypothetically no longer being feasible. Most participants further considered their current ASED therapy highly effective and rated trust in the safety and quality of serum eye drops as paramount, which is consistent with the established epitheliotrophic and biochemical properties that underpin ASED therapy [24,25]. Pronounced concerns emerged across all three assessed domains—infectious disease transmission, immunological reactions, and therapeutic efficacy—with all three correlating negatively with the willingness to switch. The strongest negative associations emerged between concerns about immunological reactions or doubts about efficacy and the general openness toward Allo-SEDs. These findings indicate that ASED users in this cohort are largely satisfied with their current therapy but remain pragmatic when faced with clinical necessity, and that perceived safety- and efficacy-related concerns constitute important barriers to the adoption of donor-derived alternatives among the domains that we assessed.

The pronounced safety and efficacy concerns expressed by participants reveal a notable discrepancy between patient perception and existing clinical evidence on donor-derived serum products [16,26]. Despite donor screening protocols comparable to those used for blood transfusions, most participants in this cohort rated their concerns about infectious disease transmission and immunological reactions as high. Multiple investigations have demonstrated comparable therapeutic efficacy, biochemical composition, and favorable safety profiles for allogeneic and autologous SEDs in established production programs [16,27,28]. Standardized aseptic processing in sealed manufacturing systems, ABO-specific blood typing, and adherence to validated production protocols have additionally minimized the residual risks associated with donor-derived eye drops [29,30]. Clinical investigations have reported comparable improvements in patient-reported outcomes and ocular surface parameters when patients receive allogeneic instead of autologous preparations [17]. A recent systematic review of 60 studies involving 1738 eyes treated with allogeneic blood-derived eye drops reported adverse events in fewer than 2% of cases, with improvements observed in patient-reported symptoms, epithelial healing, and visual outcomes [18]. Furthermore, a New Zealand cohort of 1067 patients receiving SEDs reported reactions in only 0.5% of those treated with allogeneic preparations, with no severe reactions or infections documented [21]. The discrepancy between clinical evidence and patient perception therefore likely reflects limited patient familiarity with donor screening procedures and standardized manufacturing processes rather than the actual hazard profile of Allo-SEDs. Future studies should explore whether targeted educational interventions, such as informational brochures, structured counseling sessions, or short explanatory videos addressing donor screening and manufacturing standards, can reduce safety-related reservations and improve overall acceptance of Allo-SEDs in clinical practice [31].

The pronounced concerns regarding infectious disease transmission observed in our cohort are understandable, as donor-derived serum products inherently introduce risks that are absent with autologous preparations. At the same time, these concerns are not entirely unfounded. Because allogeneic SEDs are not life-saving products, the EDQM Guide to the Quality and Safety of Tissues and Cells for Human Application (5th edition, 2022) recommends more stringent safety measures than those applied to blood products intended for transfusion [32]. Current guidance includes comprehensive infectious disease screening, product quarantine with repeat nucleic acid and/or antibody testing where appropriate, and, for pooled donor preparations, a formal risk assessment to justify pooling and minimize the risk of pathogen transmission. Importantly, even rigorous donor selection and screening cannot completely eliminate the residual risk of infection, particularly from newly emerging pathogens that may not yet be included in routine testing.

Regarding the treatment characteristics of this cohort, most participants applied diluted ASED preparations and reported a high daily application frequency. The predominance of 1:1 diluted serum preparations and frequent daily instillation in this cohort aligns with the long-term safety and effectiveness profile reported for 50% diluted ASEDs [18,33]. Patient-reported outcome data from international ASED programs demonstrate sustained symptomatic relief and improved quality of life with long-term use [6,34], which is consistent with the high perceived effectiveness participants attributed to their current therapy. When the cohort was stratified by daily application frequency, no differences in any of the three acceptance items were detected, suggesting that the perceived treatment burden alone does not appear to drive willingness to switch to Allo-SEDs. Beliefs about safety and efficacy may therefore be the dominant determinants of patient attitudes toward Allo-SEDs. Future studies should evaluate whether longer treatment durations or specific underlying diagnoses, such as graft-versus-host disease or neurotrophic keratopathy, are associated with different patterns of acceptance, and whether willingness to switch evolves over time as patients gain more information about allogeneic treatment programs.

This study has notable strengths. Of the eligible patients identified during the recruitment period, 107 of 202 (53.0%) returned the questionnaire, and a clinically heterogeneous, real-world cohort was recruited encompassing more than twenty different underlying indications that ranged from common DED to rare entities such as Stevens–Johnson syndrome and mucous membrane pemphigoid. However, we acknowledge several limitations. First, this survey-based study was conducted at a single tertiary care center in Switzerland, and the findings cannot be reliably extrapolated to a broader population, as practice patterns, regulations, and access to allogeneic serum products vary substantially between countries [35]. International surveys have documented wide variability in SED production methods, donor selection criteria, and regulatory requirements across different healthcare systems [11]. Second, none of the participants had personal experience with Allo-SEDs, and their responses therefore reflect hypothetical rather than experiential acceptance. Studies comparing patient-reported outcomes before and after actual transition from autologous to allogeneic preparations would provide more robust evidence regarding real-world acceptance [16]. Third, reliance on self-reported data without objective measures of disease severity or treatment response may have introduced recall or social desirability bias. Fourth, the questionnaire was developed specifically for this study and was not formally validated. Although the items were based on the literature and clinical experience, the absence of formal psychometric validation may limit the reliability and comparability of the responses. Moreover, the questionnaire did not capture sociodemographic factors such as educational level, and the sample was presumably underpowered for subgroup analyses. Furthermore, the demographic and clinical data describe the full eligible cohort, whereas the responses originate from the 107 patients who returned the questionnaire. Because the returns were anonymous, we could neither compare respondents with non-respondents nor demonstrate that the respondents are representative, and non-respondents may differ systematically in disease severity, treatment adherence, satisfaction, or attitudes, which could introduce non-response bias in either direction. For the same reason, we could not relate the reported attitudes to the underlying diagnosis or to the prescribed serum concentration. A further limitation concerns the information that we provided to the participants. The explanatory text accompanying the questionnaire (File S1) summarized the current evidence only briefly, stating that autologous and allogeneic SEDs show comparable efficacy and tolerability in the treatment of severe DED, that several countries have consequently moved to allogeneic preparations for practical reasons, and that serological donor screening comparable to that applied to other blood products reduces, without entirely eliminating, the risk of transmitting infectious agents. Because this summary addressed neither the varying results across studies nor the residual risk of infectious transmission in detail, it may have encouraged participants to view a switch from autologous to allogeneic preparations favorably. Finally, we assessed three pre-specified concern domains and did not capture ethical, religious, cultural, or economic reservations, so that further barriers to acceptance may exist.

## 5. Conclusions

In conclusion, this single-center survey found limited voluntary willingness among ASED users to switch to Allo-SEDs, whereas acceptance increased markedly when confronted with clinical necessity. Concerns regarding infectious disease transmission, immunological reactions, and therapeutic efficacy correlated inversely with the willingness to switch. The implementation of Allo-SED programs in Switzerland will require patient-centered education addressing safety and efficacy concerns, alongside robust donor screening and manufacturing standards. Future multicenter and longitudinal studies should evaluate patient acceptance after transition to Allo-SEDs, assess the impact of educational interventions, and compare the cost-effectiveness of autologous and allogeneic SED programs. Such studies will be essential to guide clinicians, patients, and stakeholders in the responsible introduction of allogeneic serum-based therapies in Switzerland and beyond.

## Figures and Tables

**Table 1:** The English translation of provided questionnaire. The participants were informed that they were currently receiving autologous serum eye drops prepared by the Eye Bank of the University Hospital Zurich and that participation was voluntary and anonymous. Autologous serum eye drops were described as eye drops prepared from the patient’s own blood, whereas allogeneic serum eye drops were described as eye drops prepared from the blood of healthy donors (blood group AB). Participants were informed that donor blood undergoes standard serological screening to minimise the risk of infectious disease transmission and that previous studies have reported comparable efficacy and tolerability between autologous and allogeneic serum eye drops. Response scale: 0 = does not apply at all/very poor; 5 = neutral/no opinion/don’t know; 10 = fully applies/very good.

Question	Abbreviation
Q1 How do you rate the effect of the autologous serum eye drops in your case?	Effectiveness
Q2 On average, how many times per day do you use your autologous serum eye drops?	Frequency
Q3 How important to you is trust in the safety (quality, control) of autologous or allogeneic serum eye drops?	Trust
Q4 Would you switch to allogeneic serum eye drops if they were available?	Voluntary
Q5 Would you switch to allogeneic serum eye drops if production from your own blood were no longer possible (e.g., because of anemia or difficult blood collection)?	Forced
Q6 Could you, in principle, imagine using allogeneic serum eye drops?	Consider
Q7 Do you have concerns about the transmission of infectious diseases with allogeneic serum eye drops?	Infection
Q8 How great are your concerns that allogeneic serum eye drops, because of the foreign serum, could trigger defense or immune reactions in your eyes?	Immune
Q9 Do you doubt that allogeneic serum eye drops are as effective as autologous ones?	Doubt

**Table 2:** Patient characteristics and serum dilution distribution. The 1:1 diluted serum concentration contained 50% serum and 50% balanced salt solution. These data describe the full eligible cohort of 202 ASED users retrieved from the medical records and not the 107 respondents who returned the questionnaire. SD, standard deviation; IQR, interquartile range.

Characteristic	*n* (%)
Total patients, *N*	202
Female, N (%)	136 (67.3)
Age (years)	
Mean ± SD	61.8 ± 17.5
Median (IQR)	63.5 (49.2 to 75.0)
Range	9 to 94
Treatment, years	
Mean ± SD	5.3 ± 4.9
Median (IQR)	4.0 (1.0 to 8.0)
Range	0.0 to 21.0
Serum dilution	
1:1 with balanced salt solution	140 (69.3)
Undiluted	62 (30.7)

**Table 3:** Primary diagnoses and prescribed autologous serum eye drop (ASED) concentration in the full eligible cohort of 202 patients, which does not include the 107 respondents who returned the questionnaire. Data are presented as numbers (%). Participants are categorized according to their primary treatment indication and whether they received diluted (1:1 with balanced salt solution) or undiluted (100%) ASEDs. Percentages in the diluted and undiluted columns refer to the proportion within each diagnostic category.

Primary Diagnosis	Total, *n* (%)	Diluted, *n* (%)	Undiluted, *n* (%)
Dry eye disease (DED), not otherwise specified	85 (42.1)	60 (70.6)	25 (29.4)
Sjögren’s syndrome	28 (13.9)	24 (85.7)	4 (14.3)
Graft-versus-host disease (GvHD)	20 (9.9)	17 (85.0)	3 (15.0)
Neurotrophic keratopathy after herpes keratitis (HSV-1)	9 (4.5)	4 (44.4)	5 (55.6)
Neurotrophic keratopathy after herpes zoster ophthalmicus (HZO)	8 (4.0)	4 (50.0)	4 (50.0)
Neurotrophic keratopathy, not otherwise specified	8 (4.0)	5 (62.5)	3 (37.5)
Corneal neuropathic pain (CNP)	5 (2.5)	2 (40.0)	3 (60.0)
Post-refractive surgery	5 (2.5)	5 (100.0)	0 (0.0)
Lyell’s syndrome, toxic epidermal necrolysis (TEN)	5 (2.5)	1 (20.0)	4 (80.0)
Neurotrophic keratopathy with tumor	4 (2.0)	2 (50.0)	2 (50.0)
Limbal stem cell deficiency, not otherwise specified	4 (2.0)	3 (75.0)	1 (25.0)
Chemical burn	4 (2.0)	1 (25.0)	3 (75.0)
Exposure keratopathy with facial palsy	3 (1.5)	2 (66.7)	1 (33.3)
Aniridia	3 (1.5)	2 (66.7)	1 (33.3)
Mucous membrane pemphigoid (MMP)	2 (1.0)	2 (100.0)	0 (0.0)
Basement membrane dystrophy	2 (1.0)	2 (100.0)	0 (0.0)
Neurotrophic keratopathy, trigeminal (thermocoagulation, trigeminal injury)	2 (1.0)	2 (100.0)	0 (0.0)
Exposure keratopathy, not otherwise specified	2 (1.0)	1 (50.0)	1 (50.0)
Stevens–Johnson syndrome (SJS)	1 (0.5)	1 (100.0)	0 (0.0)
Persistent epithelial defect (PED), not otherwise specified	1 (0.5)	0 (0.0)	1 (100.0)
Meesmann dystrophy (MECD)	1 (0.5)	0 (0.0)	1 (100.0)
Total	202 (100.0)	140 (69.3)	62 (30.7)

## Data Availability

The data that support the findings of this study are available from the corresponding author upon reasonable request.

## Supplementary Materials

The following supporting information can be downloaded at: https://www.mdpi.com/article/10.3390/jcm15176537/s1, File S1: Original questionnaire in the native language (German).

## Abbreviations

The following abbreviations are used in this manuscript:

Allo-SED	Allogeneic serum eye drops
ASED	Autologous serum eye drops
CI	Confidence interval
DED	Dry eye disease
EDQM	European Directorate for the Quality of Medicines & HealthCare
GvHD	Graft-versus-host disease
HLA	Human leukocyte antigen
HSV	Herpes simplex virus
HZO	Herpes zoster ophthalmicus
IQR	Interquartile range
MMP	Mucous membrane pemphigoid
OSD	Ocular surface disease
SD	Standard deviation
SED	Serum eye drops
SJS	Stevens–Johnson syndrome
TEN	Toxic epidermal necrolysis
