# Supplementary material for: Acceptance of Allogeneic Serum Eye Drops Among Users of Autologous Serum Eye Drops—A Survey-Based Study
Source: J Clin Med. 2026 Aug 24;15(17):6537. doi: 10.3390/jcm15176537 (PMC13566650; doi:10.3390/jcm15176537)
Supplement: Supplementary file 1 [file jcm-15-06537-s001.zip › jcm-4502628-supplementary File S1.pdf]

Supplementary Materials

Acceptance of Allogeneic Serum Eye Drops among Users of Autologous Serum Eye Drops – A Survey-based Study

Frank Blaser [1, *], Mohammadjavad Abbasi [2], Mario Damiano Toro [3], Timothy Hamann [1], Daniel Barthelmes [1], Sandrine Zweifel [1], Martina M Bosch [4], Isabelle Meneau [1], Jana Schneider [1], Sadiq Said [1, 4+], Vinoth Yogarasa [4+]

File S1. Original questionnaire in the native language (German)

The text below reproduces the paper questionnaire that was handed to participants during routine outpatient visits and returned by post. An English translation of the nine items is provided as Table 1 in the main manuscript, and a full English translation of the questionnaire is provided in File S2 below.

Sehr geehrte Damen und Herren,
Sie wenden Eigenserumaugentropfen (= autologe Serumaugentropfen) an, welche die Augenbank des Universitätsspitals Zürich für Sie herstellt. Wir möchten Ihnen gerne einige wenige Fragen stellen, deren Beantwortung uns bei der weiteren Ausrichtung unserer Tätigkeit zugunsten unserer PatientInnen helfen wird. Die Teilnahme ist freiwillig und die Daten werden anonymisiert behandelt.
Autologe Serumaugentropfen = Eigenserumaugentropfen = aus Ihrem eigenen Blut hergestellte Augentropfen.
Allogene Serumaugentropfen = aus Blut von gesunden Spendern hergestellte Augentropfen (mit Blutgruppe AB). Es erfolgen serologische Abklärungen der Spender, wie bei anderen Blutprodukten, um das Risiko der Übertragung von Infektionskrankheiten weitestgehend ausschliessen zu können.
Autologe und allogene Serumaugentropfen weisen in Studien eine vergleichbare Wirksamkeit und Verträglichkeit auf.
Antwort-Skala: 0 = trifft überhaupt nicht zu / sehr schlecht; 5 = neutral / keine Meinung / weiss nicht; 10 = trifft vollständig zu / sehr gut.

Fragen
Q1: Wie bewerten Sie die Wirkung der autologen Serumaugentropfen in Ihrem Fall?
Q2: Wie viele Male pro Tag benutzen Sie Ihre autologen Serumaugentropfen im Durchschnitt?
Q3: Wie wichtig ist Ihnen das Vertrauen in die Sicherheit (Qualität, Kontrolle) von autologen oder allogenen Serumaugentropfen?
Q4: Würden Sie auf allogene Serumaugentropfen umsteigen, falls diese verfügbar wären?
Q5: Würden Sie auf allogene Serumaugentropfen umsteigen, falls die Herstellung aus Ihrem eigenen Blut nicht mehr möglich ist (z. B. wegen Blutarmut oder schwieriger Blutentnahmen)?
Q6: Könnten Sie sich grundsätzlich vorstellen, allogene Serumaugentropfen zu verwenden?
Q7: Haben Sie bei allogenen Serumaugentropfen Bedenken bezüglich Übertragung von Infektionskrankheiten?
Q8: Wie gross sind Ihre Bedenken, dass allogene Serumaugentropfen durch das fremde Serum Abwehr- oder Immunreaktionen in Ihren Augen auslösen können?
Q9: Zweifeln Sie daran, dass allogene Serumaugentropfen genauso wirksam sind wie autologe?

English translation of the questionnaire

The text below is an English translation of the German questionnaire reproduced in File S1. The translation is provided for readers who do not read German; the German version in File S1 is the version that was administered to participants.

Dear Madam or Sir,

You are using own-serum eye drops (= autologous serum eye drops), which the Eye Bank of the University Hospital Zurich prepares for you. We would like to ask you a few short questions; your answers will help us to further direct our work for the benefit of our patients. Participation is voluntary, and the data are handled anonymously.

Autologous serum eye drops = own-serum eye drops = eye drops manufactured from your own blood.

Allogeneic serum eye drops = eye drops manufactured from the blood of healthy donors (with blood group AB). Donors undergo serological screening, as is done for other blood products, in order to exclude the risk of transmission of infectious diseases as far as possible.

In studies, autologous and allogeneic serum eye drops show comparable efficacy and tolerability.

Response scale: 0 = does not apply at all / very poor; 5 = neutral / no opinion / do not know; 10 = applies completely / very good.

**Questions**
Q1: How do you rate the effect of the autologous serum eye drops in your case?
Q2: On average, how many times per day do you use your autologous serum eye drops?
Q3: How important to you is trust in the safety (quality, control) of autologous or allogeneic serum eye drops?
Q4: Would you switch to allogeneic serum eye drops if these were available?
Q5: Would you switch to allogeneic serum eye drops if manufacturing from your own blood were no longer possible (e.g., because of anemia or difficult blood draws)?
Q6: In principle, could you consider using allogeneic serum eye drops?
Q7: Do you have concerns about the transmission of infectious diseases with allogeneic serum eye drops?
Q8: How great are your concerns that allogeneic serum eye drops could trigger defense or immune reactions in your eyes because of the foreign serum?
Q9: Do you doubt that allogeneic serum eye drops are as effective as autologous ones?
